# The utility of plasma circulating cell-free messenger RNA as a biomarker of glioma: a pilot study

**DOI:** 10.1007/s00701-021-05014-8

**Published:** 2021-10-13

**Authors:** Michael Itak Ita, Jiang Huai Wang, André Toulouse, Chris Lim, Noel Fanning, Michael O’Sullivan, Yvonne Nolan, George Finbarr Kaar, Henry Paul Redmond

**Affiliations:** 1 Department of Neurological Surgery, Cork University Hospital, Wilton, Cork Ireland; 2 Department of Academic Surgery, University College Cork, Cork, Ireland; 3 Department of Anatomy and Neuroscience, University College Cork, Cork, Ireland; 4 Department of Neuroradiology, University College Cork, Cork, Ireland

**Keywords:** Glioma, Biomarker, Circulating cell-free messenger RNA

## Abstract

**Background:**

Research into the potential utility of plasma-derived circulating cell-free nucleic acids as non-invasive adjuncts to radiological imaging have been occasioned by the invasive nature of brain tumour biopsy. The objective of this study was to determine whether significant differences exist in the plasma transcriptomic profile of glioma patients relative to differences in their tumour characteristics, and also whether any observed differences were representative of synchronously obtained glioma samples and TCGA glioma-derived RNA.

**Methods:**

Blood samples were collected from twenty glioma patients prior to tumour resection. Plasma ccfmRNAs and glioma-derived RNA were extracted and profiled.

**Results:**

BCL2L1, GZMB, HLA-A, IRF1, MYD88, TLR2, and TP53 genes were significantly over-expressed in glioma patients (*p* < 0.001, versus control). GZMB and HLA-A genes were significantly over-expressed in high-grade glioma patients (*p* < 0.001, versus low-grade glioma patients). Moreover, the fold change of the BCL2L1 gene was observed to be higher in patients with high-grade glioma (*p* = 0.022, versus low-grade glioma patients). There was positive correlation between the magnitude of fold change of differentially expressed genes in plasma- and glioma-derived RNA (Spearman *r* = 0.6344, *n* = 14, *p* = 0.017), and with the mean FPKM in TCGA glioma-derived RNA samples (Spearman *r* = 0.4614, *n* = 19, *p* < 0.05). There was positive correlation between glioma radiographic tumour burden and the magnitude of fold change of the CSF3 gene (*r* = 0.9813, *n* = 20, *p* < 0.001).

**Conclusion:**

We identified significant differential expression of genes involved in cancer inflammation and immunity crosstalk among patients with different glioma grades, and there was positive correlation between their transcriptomic profile in plasma and tumour samples, and with TCGA glioma-derived RNA.

**Supplementary Information:**

The online version contains supplementary material available at 10.1007/s00701-021-05014-8.

## Introduction

Investigations into the utility of plasma-derived circulating cell-free nucleic acids as potential non-invasive adjuncts to radiological imaging have been occasioned by the invasive nature of brain tumour tissue biopsy. Gliomas are the most common primary brain malignancies in adults [10]. Glioblastoma (GBM) accounts for more than 50% of all diagnosed cases of glioma [20, 30]. This class of glioma is further categorised into proneural, mesenchymal, and classical subtypes based on the findings of proteomic, transcriptomic, and genomic profiling [21]. Moreover, this categorisation of glioblastoma into molecular subtypes has important implications for therapeutic response and prognosis [21]. While the therapeutic strategies for high-grade glioma have progressed to encompass the applications of the STUPP protocol (i.e., maximal resection of glioblastoma, followed by concomitant radiotherapy, and chemotherapy with the oral alkylating agent temozolomide) [24], combination therapy with lomustine-temozolomide [4], fluorescence-guided surgery (FGS) using 5-aminolevulinic acid (5-ALA) [5], and NovoTTF-100A device in combination with temozolomide [7]; the mortality rate associated with high-grade glioma remains high [22]. Radiological imaging studies and tumour tissue biopsy currently constitute the established modalities for diagnosing glioblastoma, and for surveillance in affected patients [25]. However, differentiating between actual malignant tumour progression and the event of pseudoprogression remains a radiological imaging challenge [19]. Furthermore, the problems of tumour heterogeneity and evolving glioma genomic landscape hamper the essence of tumour tissue biopsy [27]. Biological fluids including blood and cerebrospinal fluid (CSF) harbour elements such as circulating tumour cells and extracellular vesicles containing proteins and cell-free nucleic acids (cfNAs) originating from malignant tumours occurring within a host [17, 18]. These circulating tumoural elements have the potential to provide insight into the biological status or characteristics of malignant tumours and, therefore, constitute the basis for liquid biopsy [19]. Minimally invasive strategies, i.e., blood and CSF sampling, offer the potential for both static and longitudinal characterisation of malignant tumours.

Circulating cell-free messenger ribonucleic acids (ccfmRNAs) are short fragments of RNA present in blood. The presence of tumour-related RNA in the plasma and serum samples of patients with cancer has been previously reported [2, 9, 13]. A number of studies have demonstrated that despite their lability, the levels of ccfmRNAs in plasma are higher in cancer patients compared to healthy persons [3, 9, 26]. The objective of this study was to determine whether significant differences exist in the plasma transcriptomic profile of glioma patients relative to differences in their tumour characteristics, i.e., glioma grade or tumour type. Moreover, we sought to determine whether any observed differences in the plasma transcriptomic profile of patients with glioma were representative of the profile of synchronously obtained glioma tumour tissue and TCGA glioma-derived RNA samples.

## Materials and methods

### Patients and samples

We initiated a prospective serial biomarker cohort study at Cork University Hospital Ireland, Department of Neurological Surgery in March 2019. Ethical approval (ECM 4 (aa). 120219) for this project was granted by the Clinical Research Ethics Committee, University College Cork Teaching Hospitals in February 2019. Adult patients with radiographically suspected high-grade glioma were eligible for enrolment.

### Extraction of plasma ccfmRNA

Participating patients had their blood samples collected in VACUETTE® TUBE 3 ml K3E K3EDTA collection tubes (Greiner Bio-One, Frickenhausen, Germany) prior to surgical resection or biopsy of their brain tumours. All blood samples were centrifuged (Centrifuge 5810 R, Eppendorf, Hamburg, Germany) at a temperature of 4 °C for 10 min at 3000 rpm to separate plasma. Patients’ plasma samples were subsequently frozen (Haier Bio-Medical, Qingdao, China) and stored at − 80 °C. RNA was extracted from patients’ plasma samples using miRNeasy Serum/Plasma Kit (Qiagen, Hilden, Germany) according to the RNA extraction protocol provided by the manufacturers, and total RNA was extracted from glioma samples using RNeasy Mini Kit (Qiagen, Hilden, Germany). Complementary DNA (cDNA) was synthesised from RNA using RT^2^ First Strand Kit (Qiagen, Hilden, Germany) according to manufacturer’s instructions.

### Data analysis

#### Gene expression analysis

Complementary DNA derived from RNA was profiled. Pathway-focused gene expression analysis was performed using RT^2^ Profiler PCR Array (Cat. no. 330231 PAHS-181Z) (Qiagen, MD, USA) in combination with RT^2^ SYBR® Green qPCR Master-mix (Cat. no. 330529) (Qiagen, MD, USA). This PCR array was set up according to the manufacturer’s instructions. Although eighty-four genes implicated in human cancer inflammation and immunity crosstalk were profiled, only forty-three amplifiable circulating cell-free messenger RNA gene transcripts were identified in the plasma samples of enrolled patients. Gene expression data was analysed using the Qiagen Gene Globe data analysis tool which uses Student’s *t*-test of the replicate 2^ (–Delta CT) values for each of the genes being profiled in the control and test groups. The calculation of the *p*-value was based on parametric, unpaired, two-sample equal variance, two-tailed distribution. The Bonferroni procedure was used to limit family-wise error rate: (*p* < *a* / *m*), where the *p*-value (*p*) in the equation is less than or equal to the ratio of alpha (*a*), i.e., 0.05, to the number of hypotheses (*m*) being tested, i.e., 43; *p* ≤ 0.0012. All samples passed PCR array reproducibility test, reverse transcription efficiency test, and genomic DNA contamination test. Normalisation was performed using the RPLP0 gene. This gene was automatically selected from a panel of house-keeping genes (which also included ACTB, B2M, GAPDH, and HPRT1).

Correlation data analysis and descriptive statistical analysis were performed using GraphPad Prism software (Version 8.0.1 (244), San Diego, CA, USA); the correlation assessment tool Spearman’s ranks correlation test was used to evaluate for correlation between the magnitudes of gene fold change in plasma and tissues, as well as the average fragments per kilobase of transcript per million mapped reads (FPKM) of glioma tumour tissue RNA samples derived from the cancer genome atlas (https://www.cancer.gov/tcga).

### Tumour burden estimation

Magnetic resonance imaging (MRI) study was used for radiographic tumour burden estimation. This was undertaken by consultant neuroradiologists. Radiographic tumour burden was calculated using the formula for ellipsoid volumes (tumour volume = 4/3 * π * *a* * *b* * c / 8), where *a*, *b*, and *c* in the equation represent diameters in the *x*, *y*, and *z* axes.

## Results

Twenty patients with radiographically suspected GBM were enrolled in this study. High-grade glioma (HGG) was confirmed by histology in sixteen patients and four patients were diagnosed with low-grade glioma (LGG); there were a total of twenty glioma patients, see Table 1. Glioblastoma multiforme was diagnosed in a total of fifteen patients with high-grade glioma, and one patient was diagnosed with anaplastic astrocytoma. Pilocytic astrocytoma was diagnosed in two patients with low-grade glioma, and two other patients were diagnosed with diffuse astrocytoma, see Table 2. There were seven healthy controls.

**Table 1:** Patient characteristics

Variables	Glioma_median (range) total = 20	Percentage (%)
Glioma patients_gender F:M	6:14	30:70
Healthy controls_gender F:M	3:4	43:57
Glioma patients_age (years)	58 (36–70)	
Healthy controls_age (years)	39 (34–87)	
Histology of glioma		
Glioblastoma (WHO grade IV)	15/20	75
Anaplastic astrocytoma (WHO grade III)	1/20	5
Diffuse astrocytoma (WHO grade II)	2/20	10
Pilocytic astrocytoma (WHO grade I)	2/20	10
Radiographic volume of glioma (mm^3)	22.6 (4.13–161.4)

**Table 2:**
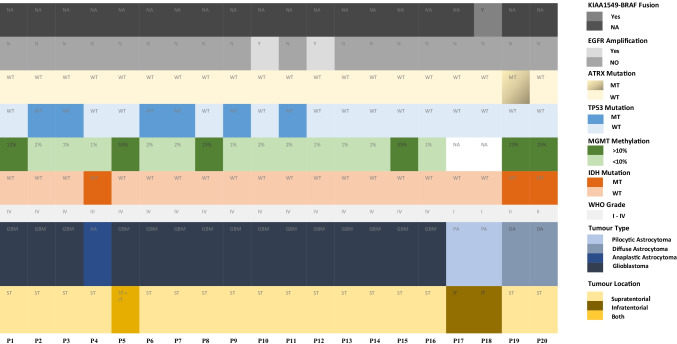
Clinical characteristics

### Differences in the gene expression profile between glioma patients and healthy controls using plasma ccfmRNAs

**Fig. 1:**
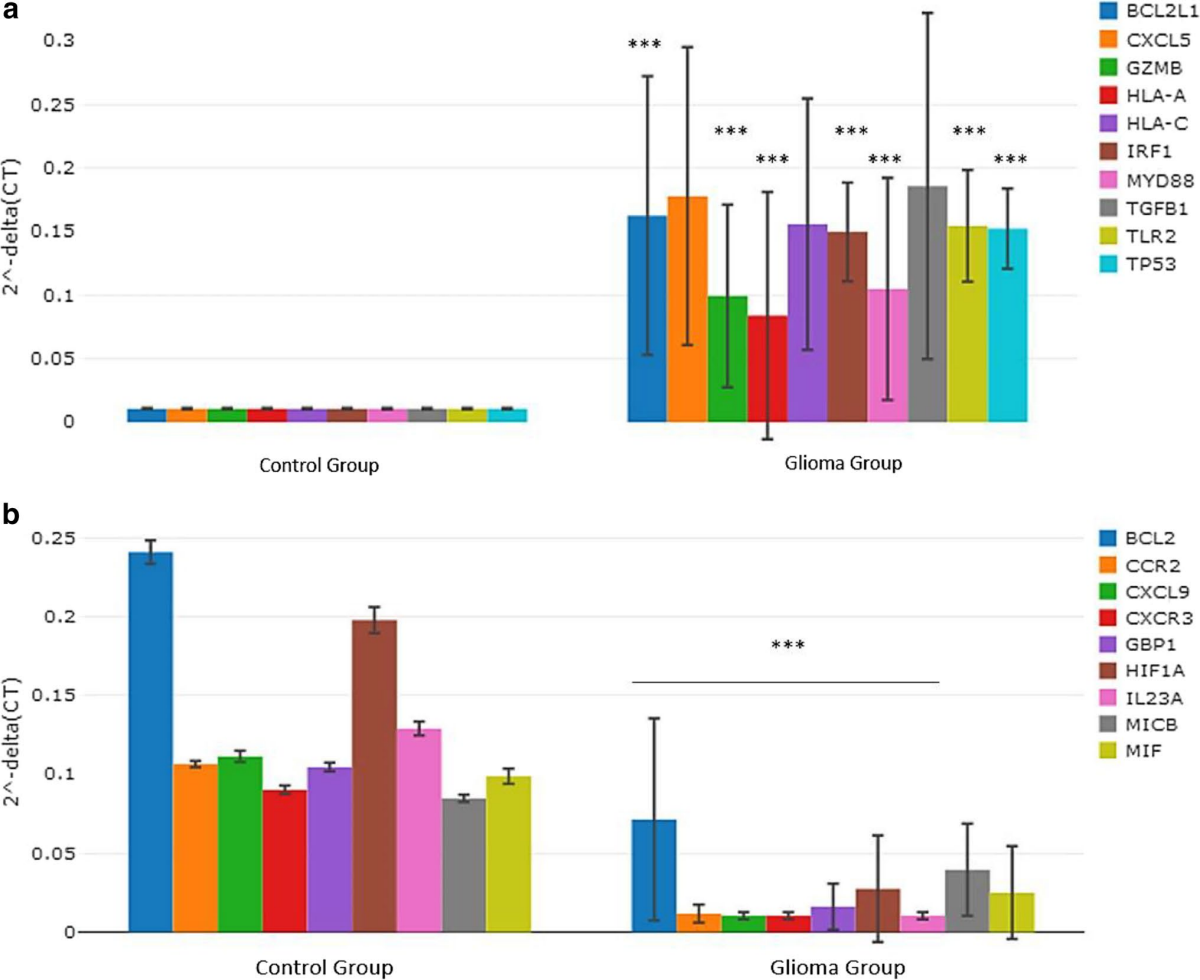
**a** Column chart demonstrating significantly over-expressed genes in the plasma samples of glioma patients relative to healthy control. **b** Column chart demonstrating significantly under-expressed genes in the plasma samples of glioma patients relative to healthy control

When the plasma transcriptomic profile of glioma patients (i.e., high-grade glioma and low-grade glioma patients) was compared to healthy controls, the following genes were observed to be significantly over-expressed: BCL2L1, GZMB, HLA-A, IRF1, MYD88, TLR2, and TP53 (*p* < 0.001, versus control), see Fig. 1a. Moreover, the magnitudes of fold changes of the following genes were observed to be higher in the glioma group compared to the control group: CXCL5 (*p* = 0.018), HLA-C (*p* = 0.014), and TGFB1 (*p* = 0.020), see Fig. 1a. BCL2, CCR2, CXCL9, CXCR3, GBP1, HIF1A, and IL23A genes were significantly under-expressed in glioma patients (*p* < 0.001, versus control), see Fig. 1b. The fold changes of the MICB (*p* = 0.038) and MIF (*p* = 0.012) genes were observed to be lower in the glioma group relative to the control group.

### Differences in the gene expression profile between high-grade glioma and low-grade glioma patients using plasma ccfmRNAs

**Fig. 2:**
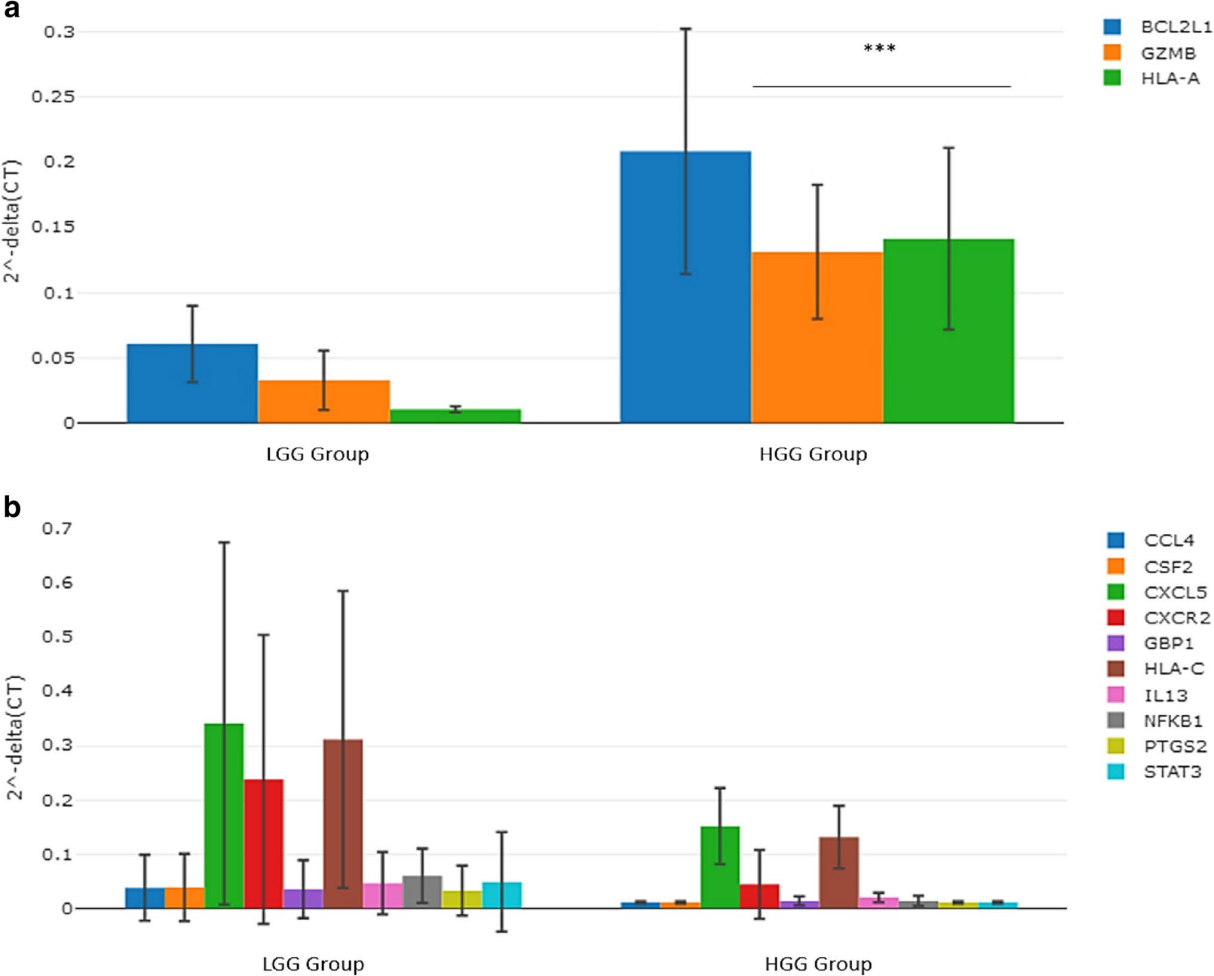
**a** Column chart demonstrating significantly over-expressed genes in the plasma samples of high-grade glioma patients relative to low-grade glioma patients. **b** Column chart demonstrating under-expressed genes in the plasma samples of high-grade glioma patients relative to low-grade glioma patients

We then compared the plasma transcriptomic profile of patients with high-grade glioma to patients with low-grade glioma. GZMB (*p* < 0.001) and HLA-A (*p* < 0.001) genes were observed to be significantly over-expressed in high-grade glioma patients relative to low-grade glioma patients, see Fig. 2a. Moreover, the magnitude of fold change of the BCL2L1 (*p* = 0.022) gene was observed to be higher in patients with high-grade glioma compared to patients with low-grade glioma, see Fig. 2a. The magnitudes of fold change of CCL4 (*p* = 0.004), CSF2 (*p* = 0.004), CXCL5 (*p* = 0.007), CXCR2 (*p* = 0.018), GBP1 (*p* = 0.014), HLA-C (*p* = 0.006), IL13 (*p* = 0.009), NFKB1 (*p* = 0.005), PTGS2 (*p* = 0.004), and STAT3 (*p* = 0.003) genes were observed to be lower in high-grade glioma patients compared to low-grade glioma patients, see Fig. 2b.

### Differences in the gene expression profile between high-grade glioma and low-grade glioma patients using RNAs derived from glioma samples

**Fig. 3:**
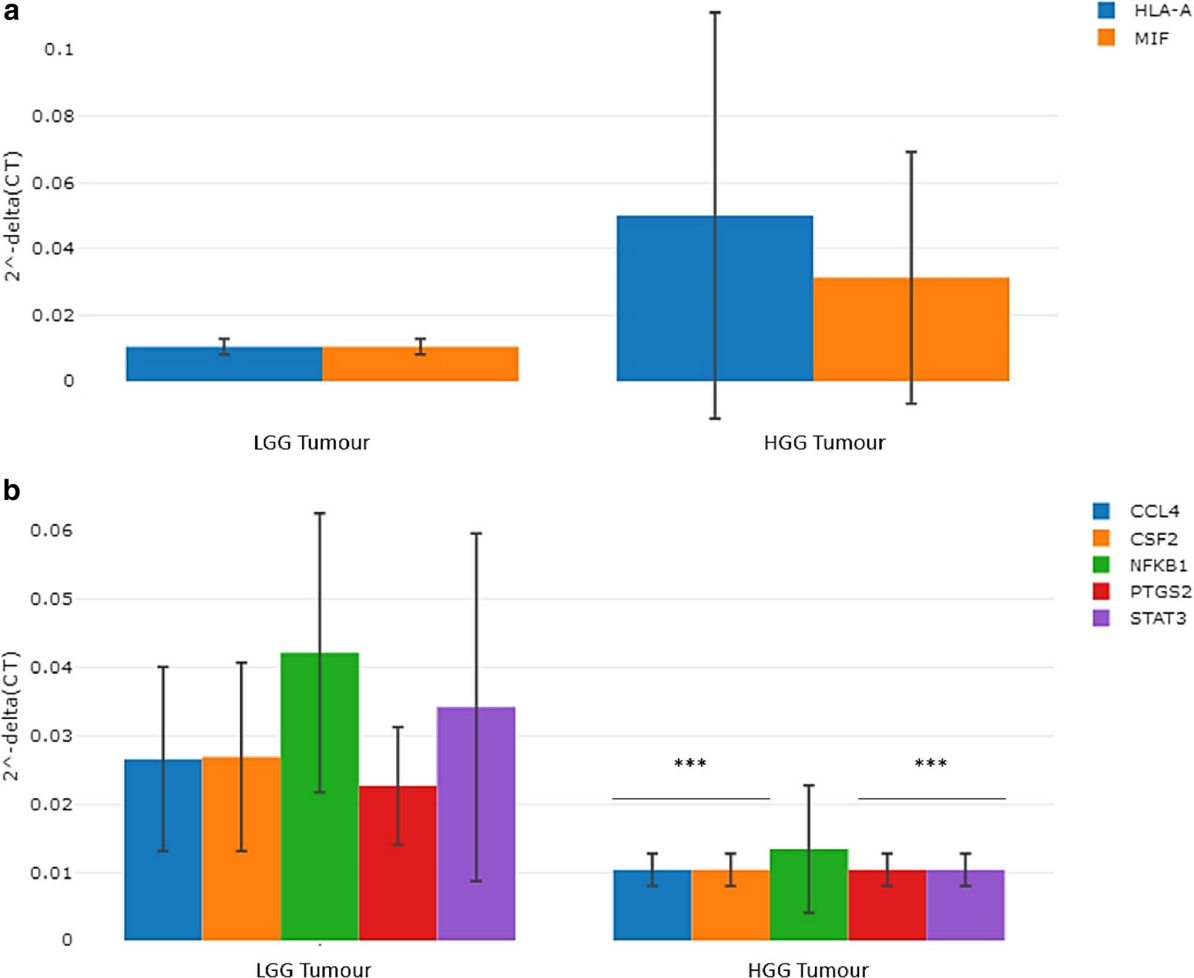
**a** Column chart demonstrating relatively over-expressed genes in the tumour of high-grade glioma patients relative to low-grade glioma patients. **b** Column chart demonstrating significantly under-expressed genes in the tumour samples of high-grade glioma patients relative to low-grade glioma patients

The transcriptomic profile of the total RNA derived from the tumour samples of high-grade glioma patients was compared to those derived from the tumour samples of low-grade glioma patients. The magnitudes of fold change of the HLA-A (*p* = 0.08) and MIF (*p* = 0.10) genes were observed to be relatively higher in the tumour samples of high-grade glioma patients compared to low-grade glioma patients although this observation did not attain to a level of statistical significance, see Fig. 3a. Moreover, the following genes: CCL4, CSF2, PTGS2, and STAT3 were observed to be significantly under-expressed in the tumour samples of high-grade glioma patients (*p* < 0.001, versus low-grade glioma tumour samples), see Fig. 3b. The magnitude of fold change of the NFKB1 (*p* = 0.037) gene was lower in the tumour samples of high-grade glioma patients compared to low-grade glioma patients, see Fig. 3b.

### Comparison of differentially expressed genes in glioma patient plasma samples to the magnitude of fold change of genes in patient tumour samples

**Fig. 4:**
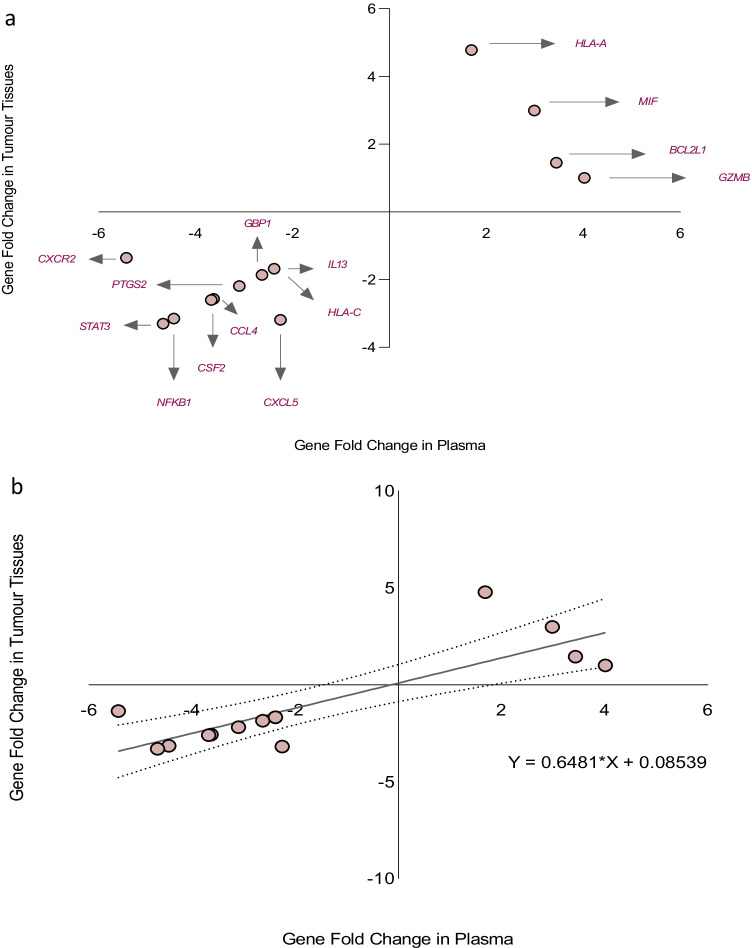

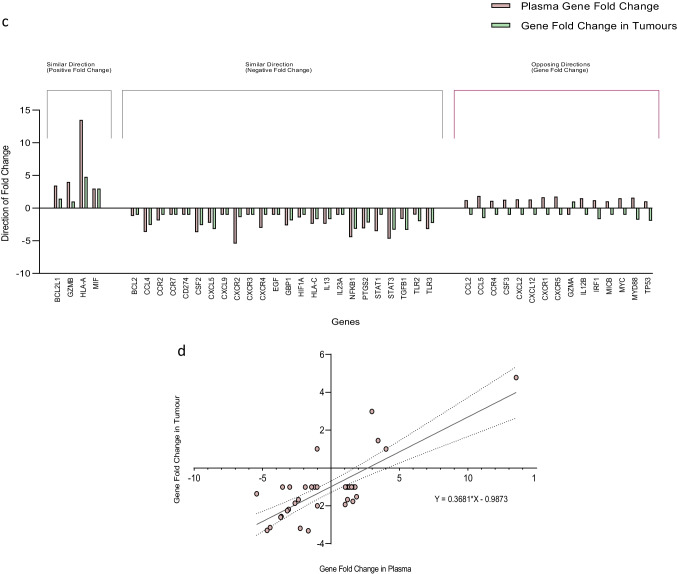
**a** Data plot demonstrating positive correlation between the magnitude of fold change of differentially expressed genes in patient plasma samples and the magnitude of fold change of glioma-derived RNA samples (Spearman *r* = 0.6344, *n* = 14, *p* = 0.017). **b** Simple linear regression demonstrating a significant relationship between the gene fold changes in plasma and tumour tissue. **c** Data plot demonstrating the direction of gene fold change of all amplifiable cell-free messenger RNA transcripts identified in patient plasma and RNA in tumour samples. **d** Data plot demonstrating positive correlation between the magnitude of fold change of all amplifiable cell-free messenger RNA transcripts in patient plasma samples and the magnitude of fold change of glioma-derived RNA samples (Spearman *r* = 0.6257, *n* = 43, *p* < 0.001)

We then evaluated for association between the magnitudes of fold change of differentially expressed genes in the plasma samples of high-grade glioma patients relative to low-grade glioma patients and the genes expressed in patient tumour samples (i.e., high-grade glioma tumour samples relative to low-grade glioma tumour samples). There was positive correlation between the magnitudes of fold change of differentially expressed genes in patient plasma samples and the magnitudes of fold change of genes expressed in patient tumour samples: BCL2L1, CCL4, CSF2, CXCL5, CXCR2, GBP1, GZMB, HLA-A, HLA-C, IL13, MIF, NFKB1, PTGS2, and STAT3 (Spearman *r* = 0.6344, *n* = 14, *p* = 0.017), see Fig. 4a. Simple linear regression analysis demonstrated a significant relationship between the gene fold changes in plasma and tumour tissue: *Y* = 0.6481 * *X* + 0.08539, *p* < 0.001, see Fig. 4b. A total of forty-three amplifiable cell-free messenger RNA transcripts were identified in patient plasma samples, and fourteen of these genes were observed to be differentially expressed. When comparing the plasma transcriptomic profile of high-grade glioma patients to low-grade glioma patients, the magnitudes of fold change of 65.1% (28/43) of all amplifiable gene transcripts in plasma were observed to occur in a direction similar to the gene fold change in patient tumour samples (i.e., high-grade glioma tumour samples relative to low-grade glioma tumour samples), see Fig. 4c. Moreover, there was correlation between all the amplifiable cell-free messenger RNA transcripts observed in patient plasma samples and the genes expressed in tumour tissues (Spearman *r* = 0.6257, *n* = 43, *p* < 0.001); with a significant regression equation: *Y* = 0.3681 * *X* − 0.9873, *p* < 0.001, see Fig. 4d.

### Comparison of differentially expressed genes in glioma patient plasma samples with the average FPKM in TCGA glioma-derived RNA samples

**Fig. 5:**
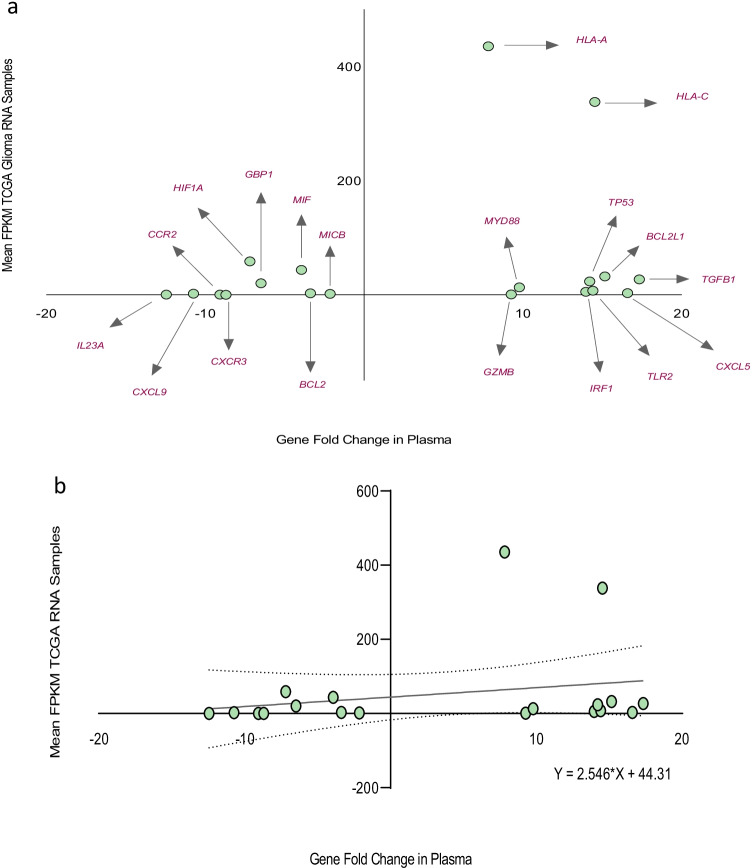
**a** Data plot demonstrating positive correlation between the magnitude of fold change of differentially expressed genes in the plasma samples of glioma patients and the mean FPKM of TCGA glioma-derived RNA samples (Spearman *r* = 0.4614, *n* = 19, *p* = 0.047). **b** Simple linear regression did not demonstrate a significant relationship between the gene fold changes in plasma and the mean FPKM of TCGA glioma-derived RNA samples

Furthermore, we assessed for potential association between the magnitudes of fold change of differentially expressed genes in the plasma samples of all glioma patients relative to healthy controls and the average FPKM in TCGA glioma-derived RNA samples. There was positive correlation between the magnitudes of fold change of differentially expressed genes in glioma patient plasma samples and the mean FPKM in TCGA glioma-derived RNA samples: BCL2, BCL2L1, CCR2, CXCL5, CXCL9, CXCR3, GBP1, GZMB, HIF1A, HLA-A, HLA-C, IL23A, IRF1, MICB, MIF, MYD88, TGFB1, TLR2, and TP53; Spearman *r* = 0.4614, *n* = 19, *p* = 0.047, see Fig. 5a. However, simple linear regression analysis did not demonstrate a significant relationship between the fold changes of differentially expressed genes in plasma and the average fragments per kilobase of transcript per million mapped reads in TCGA glioma-derived RNA samples: *Y* = 2.546 * *X* + 44.31, *p* = 0.336, see Fig. 5b. Forty-three amplifiable cell-free messenger RNA transcripts were observed in patient plasma samples, and a total of nineteen of these genes were differentially expressed. When comparing the plasma transcriptomic profile of all glioma patients (i.e., both high-grade glioma and low-grade glioma patients relative to healthy controls) to the average fragments per kilobase of transcript per million mapped reads in TCGA glioma-derived RNA samples (FPKM values less than the median < 3.1 were considered to be low, and FPKM values greater than the median > 3.1 were considered to be high), the magnitudes of gene fold change of 67.4% (29/43) of these amplifiable cell-free messenger RNA transcripts in patient plasma samples were observed to occur in a direction similar to the average FPKM in TCGA glioma-derived RNA samples, see supplementary Fig. 1a. However, there was no significant correlation between all the amplifiable cell-free messenger RNA transcripts observed in patient plasma samples and the average fragments per kilobase of transcript per million mapped reads in TCGA glioma-derived RNA samples, Spearman *r* = 0.2514, *n* = 43, *p* = 0.104; with a non-significant regression equation: *Y* = 1.925 * *X* + 23.81, *p* = 0.181, see supplementary Fig. 1b.

### Correlation analysis demonstrating positive correlation between glioma tumour burden and the magnitude of fold change of the CSF3 gene

**Fig. 6:**
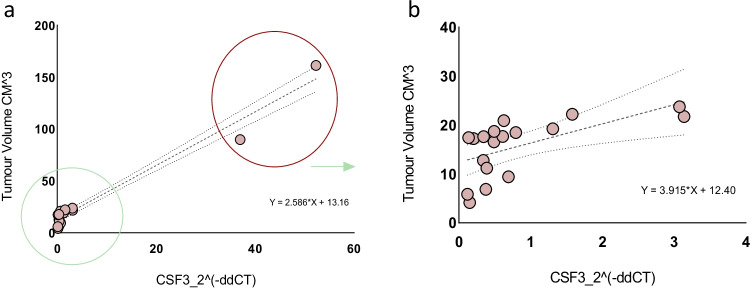
**a** Data plot demonstrating positive correlation between glioma radiographic tumour burden and the magnitude of fold change of CSF3 gene (*r* = 0.9813, *n* = 20, *p* < 0.001). **b** Positive correlation between glioma radiographic tumour burden and the magnitude of fold change of the CSF3 gene (*r* = 0.6078, *n* = 18, *p* = 0.007) despite exclusion of outliers

We assessed for potential association between the magnitudes of fold change of all amplifiable cell-free messenger RNA transcripts identified in the plasma samples of all glioma patients relative to health controls and radiographic tumour burden. There was a significant positive correlation (*r* = 0.9813, *n* = 20, *p* < 0.001) between glioma radiographic tumour burden and the magnitude of fold change of the CSF3 gene in the plasma samples of glioma patients (relative to the control group), see Fig. 6a; this association between the magnitude of fold change of the CSF3 gene in plasma and radiographic tumour burden persisted (*r* = 0.6078, *n* = 18, *p* = 0.007) despite the exclusion of the outliers, see Fig. 6b. There was no correlation between radiographic tumour burden and the magnitude of fold change of other genes in the plasma samples of glioma patients: BCL2 (*p* = 0.840), BCL2L1 (*p* = 0.404), CCL2 (*p* = 0.859), CCL4 (*p* = 0.686), CCL5 (*p* = 0.667), CCR2 (*p* = 0.965), CCR4 (*p* = 0.993), CCR7 (*p* = 0.979), CD274 (*p* = 0.979), CSF2 (*p* = 0.682), CXCR1 (*p* = 0.851), CXCR2 (*p* = 0.247), CXCR3 (*p* = 0.979), CXCR4 (*p* = 0.215), CXCR5 (*p* = 0.067), CXCL2 (*p* = 0.848), CXCL5 (*p* = 0.456), CXCL9 (*p* = 0.979), CXCL12 (*p* = 0.499), EGF (*p* = 0.979), GBP1 (*p* = 0.658), GZMA (*p* = 0.979), GZMB (*p* = 0.221), HIF1A (*p* = 0.414), HLA-A (*p* = 0.710), HLA-C (*p* = 0.203), IL12B (*p* = 0.712), IL13 (*p* = 0.398), IL23A (*p* = 0.979), IRF1 (*p* = 0.060), MICB (*p* = 0.524), MIF (*p* = 0.430), MYC (*p* = 0.099), MYD88 (*p* = 0.333), NFKB1 (*p* = 0.630), PTGS2 (*p* = 0.780), STAT1 (*p* = 0.799), STAT3 (*p* = 0.566), TGFB1 (*p* = 0.326), TLR2 (*p* = 0.301), TLR3 (*p* = 0.793), and TP53 (*p* = 0.517), see supplementary Figs. 1c-d.

## Discussion

Genomic profiling of cell-free nucleic acids is increasingly emerging as an invaluable tool for precision oncology. Investigations into the potential utility of circulating cell-free messenger RNA as a non-invasive biomarker of cancer have been hampered by concerns relating to the stability of circulating RNA, and the presence of RNases in plasma [6], which presumably contribute to the fragmentation and early degradation of circulating RNA. Circulating nucleic acid biomarker research has mainly focused on cell-free DNA, circulating tumour DNA, and micro RNA, with only limited studies on cell-free messenger RNA. In this study, we evaluated the plasma transcriptomic profile of glioma patients with a focus on genes involved in cancer inflammation and immunity crosstalk using plasma ccfmRNAs.

We identified significant differential expression of this class of genes among patients with glioma using complementary DNA derived from plasma ccfmRNAs. When we compared the plasma transcriptomic profile of glioma patients to healthy controls, a total of fourteen genes were found to be differentially expressed. Moreover, when the plasma transcriptomic profile of patients with high-grade glioma was compared to patients with low-grade glioma, the GZMB and HLA-A genes were observed to be significantly over-expressed in the high-grade glioma group relative to the low-grade glioma group. Furthermore, the magnitude of fold change of the BCL2L1 gene was observed to be higher in patients with high-grade glioma compared to patients with low-grade glioma. A total of ten genes including CCL4, CSF2, CXCL5, CXCR2, GBP1, HLA-C, IL13, NFKB1, PTGS2, and STAT3 were observed to be under-expressed in the plasma samples of patients with high-grade glioma relative to low-grade glioma patients. Furthermore, a comparison of the magnitude of fold change of differentially expressed genes in glioma patient plasma samples to those in glioma tumour samples resulted in positive correlation between the transcriptomic profiles observed in plasma and in the glioma tumour samples. When the magnitude of fold change of differentially expressed genes in glioma patient plasma samples (relative to healthy controls) was compared with the average FPKM in TCGA glioma-derived RNA samples, a weak but significant positive correlation was observed between the plasma transcriptomic profile of glioma patients and the FPKM of TCGA glioma-derived RNA samples. Although we did not observe a significant correlation between the magnitudes of fold change of all amplifiable cell-free messenger RNA transcripts (i.e., both differentially expressed and non-differentially expressed ccfmRNA transcripts) identified in plasma and the FPKM in TCGA glioma-derived RNA samples, the magnitudes of fold change of up to 67.4% of these plasma ccfmRNA transcripts in glioma patients were observed to occur in a direction similar to the average FPKM in TCGA glioma-derived RNA samples. We further evaluated for possible correlation or association between the magnitudes of fold change of the various genes involved in cancer inflammation and immunity crosstalk and radiographic tumour burden. We identified positive correlation between glioma tumour burden and the magnitude of fold change of the CSF3 gene in plasma, suggesting a potential role for this ccfmRNA transcript as a possible biomarker of tumour burden.

The clinical relevance of these observed differentially expressed genes in the pathogenesis of glioma will require further detailed studies. We discuss here based on the current knowledge, and relevant peer-reviewed scientific literature, the potential significance of the over-expressed genes observed in the plasma samples of glioma patients. The BCL2L1 gene was observed to be significantly over-expressed in the plasma samples of glioma patients relative to healthy controls, and its fold change was observed to be relatively higher in the plasma samples of patients with high-grade glioma compared to patients with low-grade glioma. Adach-Kilon et al. previously demonstrated the event of an increase in the expression of the BCL2L1 gene in proliferating glioma cells [1]. The increase in the expression of this anti-apoptotic pro-survival gene is thought to be mediated by STAT1 [1]. Kim et al. using a mathematical model were able to predict a decrease in the growth rate of residual glioma cells exposed to therapeutic agents targeting CXCL5, a member of the CXC subfamily of chemokines with an important function in tumour angiogenesis [8]. The fold change of the CXCL5 gene was observed to be relatively higher in the plasma samples of glioma patients compared to healthy controls. The granzyme B gene encodes the GZMB protein which is a serine protease belonging to the peptidase S1 family; over-expression of this serine protease has been previously shown to be associated with poor survival outcomes in patients with glioblastoma [23]. The GZMB gene was observed to be significantly over-expressed in the plasma samples of high-grade glioma patients compared to low-grade glioma patients. The HLA-A gene was observed to be significantly over-expressed in the plasma samples of glioma patients relative to the control group, and its fold regulation was observed to be significantly higher in the plasma samples of high-grade glioma patients compared to low-grade glioma patients. The fold change of the HLA-C gene was observed to be relatively higher in the plasma samples of glioma patients compared to healthy controls. Evaluation of possible associations between glioma and human leucocyte antigens (HLAs) undertaken by Machulla et al. identified significant correlations of single HLAs and symptomatic glioma in adult patients [14]. Liang et al. demonstrated that the depletion of interferon regulatory factor 1 encoded by the IRF1 gene increased the efficacy of bevacizumab (anti-VEGF-A) treatment in a glioma xenograft model through enhanced apoptosis-inducing factor-dependent apoptosis [12]. The IRF gene was observed to be significantly over-expressed in glioma patients. The MYD88 gene was significantly over-expressed in the plasma samples of glioma patients; a depletion or deficiency of this cytosolic adapter protein has been previously shown to attenuate the expansion of glioma in animal studies [15]. The magnitude of fold change of the TGFB1 protein was observed to be higher in the plasma samples of glioma patients relative to healthy controls; this protein was shown to be a target of chemokine receptor 7 (CCR7) by Zheng et al. [31]. Their study demonstrated that CCR7 mediates TGFB1-induced migration of glioma cells through the activation of matrix metalloproteinase 2 (MMP2)/9 [31]. The TLR2 gene was significantly over-expressed in the plasma samples of glioma patients. Chenglong et al. demonstrated that TLR2, a member of the Toll-like receptor family, promotes the development and progression of glioma by enhancing autophagy [11]. Synthesis of cytochrome C oxidase 2 (SCO2) gene is a target of TP53 [16, 28]. This TP53 target is capable of prolonging cancer cell survival through the activation of oxidative phosphorylation in cancer cells which ultimately results in a delay in glucose depletion [28]; the fold regulation of the TP53 gene was observed to be significantly higher in the plasma samples of patients with glioma. The C12orf5 gene encodes the protein TP53-induced glycolysis regulatory phosphatase (TIGAR); Wanka et al. demonstrated that the expression of TIGAR protected TP53 mutant cells from death under the conditions of severe hypoxia [29].

While the genomic features of the plasma circulating cell-free messenger RNA of glioma patients may not have unequivocally reflected the exact pattern of gene expression obtainable in the tumour tissue samples in this pilot study, the observation of positive correlation between the magnitudes of fold change of amplifiable messenger RNA transcripts in the plasma and synchronously obtained tumour tissue samples of glioma patients (including the correlation with the fragments per kilobase of transcript per million mapped reads of glioma-derived messenger RNA in the TCGA database) appears to strengthen the case for bodily fluids functioning as potential tumour biomarker repositories in glioma patients. Indeed, circulating oncological biomarker analysis is predicated on the assumption that perfused malignant tumour tissues within a host shed their tumoural contents into the extracellular space and consequently modify the content of bodily fluids including plasma, which may have utility as a minimally invasive or easily accessible milieu for obtaining information on the transcription dynamics occurring within the tumour or within the tumour micro-environment in patients with glioma. Moreover, the observations of significant differences between the plasma transcriptomic profile of glioma patients and healthy persons, as well as the differences between the circulating transcriptomic profile of high-grade glioma and low-grade glioma patients, may in due course lend itself to exploitation for the purpose of glioma diagnosis and characterisation, and, furthermore, for pharmacogenomic analysis in the course of glioma patient treatment. These observed differences and associations relating to the plasma transcriptomic profile of glioma patients may be of clinical importance if validated in a larger study cohort.

In conclusion, we observed significant differential expression of various genes involved in cancer inflammation and immunity crosstalk using ccfmRNAs derived from the plasma samples of patients with different glioma grades, and we identified positive correlation between the plasma transcriptomic profile of these patients and the fold changes of genes in synchronously obtained tumour samples, and with the FPKM of TCGA glioma-derived RNA samples. Our research endeavour is now focused on the utility of these blood borne ribonucleic acid transcripts as potential biomarkers for serial monitoring of therapeutic response in glioma patients and in disease prognostication.

## Limitations

Although this study was powered to reflect the effect size seen, the sample size was relatively small. Moreover, pathway-focused gene expression analysis was undertaken with a focus on genes involved in cancer inflammation and immunity crosstalk, and only eighty-four genes were profiled. The authors were not blinded to the histology of the tumours.

## Supplementary Information

Below is the link to the electronic supplementary material.

Supplementary file 1 Supplementary figure 1a-b. (a) Data plot demonstrating the direction of fold change of amplifiable ccfmRNA transcripts in plasma samples and the mean FPKM of TCGA glioma derived RNA samples (FPKM values less than the median < 3.1 were considered to be low, and FPKM values greater than the median > 3.1 were considered to be high). (b) Data plot demonstrating no correlation between the magnitude of fold change of all amplifiable cell free messenger RNA transcripts identified in the plasma samples of glioma patients and the mean FPKM of TCGA glioma derived RNA samples (Spearman r=0.2514, n=43, p=0.104). (PPTX 49 KB)

Supplementary file 2 (PPTX 54 KB)

Supplementary file 3 Supplementary figure 1c-d. Data plot showing no correlation between glioma radiographic tumour burden and the magnitude of fold change of amplifiable cell free messenger RNA transcripts identified in the plasma samples of glioma patients. (PPTX 197 KB)

Supplementary file 4 (PPTX 82 KB)

## Data Availability

The data that support the findings of this study are available from the corresponding author upon reasonable request.
