# Supplementary figures and images for: The utility of plasma circulating cell-free messenger RNA as a biomarker of glioma: a pilot study
Source: Acta Neurochir (Wien). 2021 Oct 13;164(3):723–35. doi: 10.1007/s00701-021-05014-8 (PMC8913523; doi:10.1007/s00701-021-05014-8)

## Slide 1
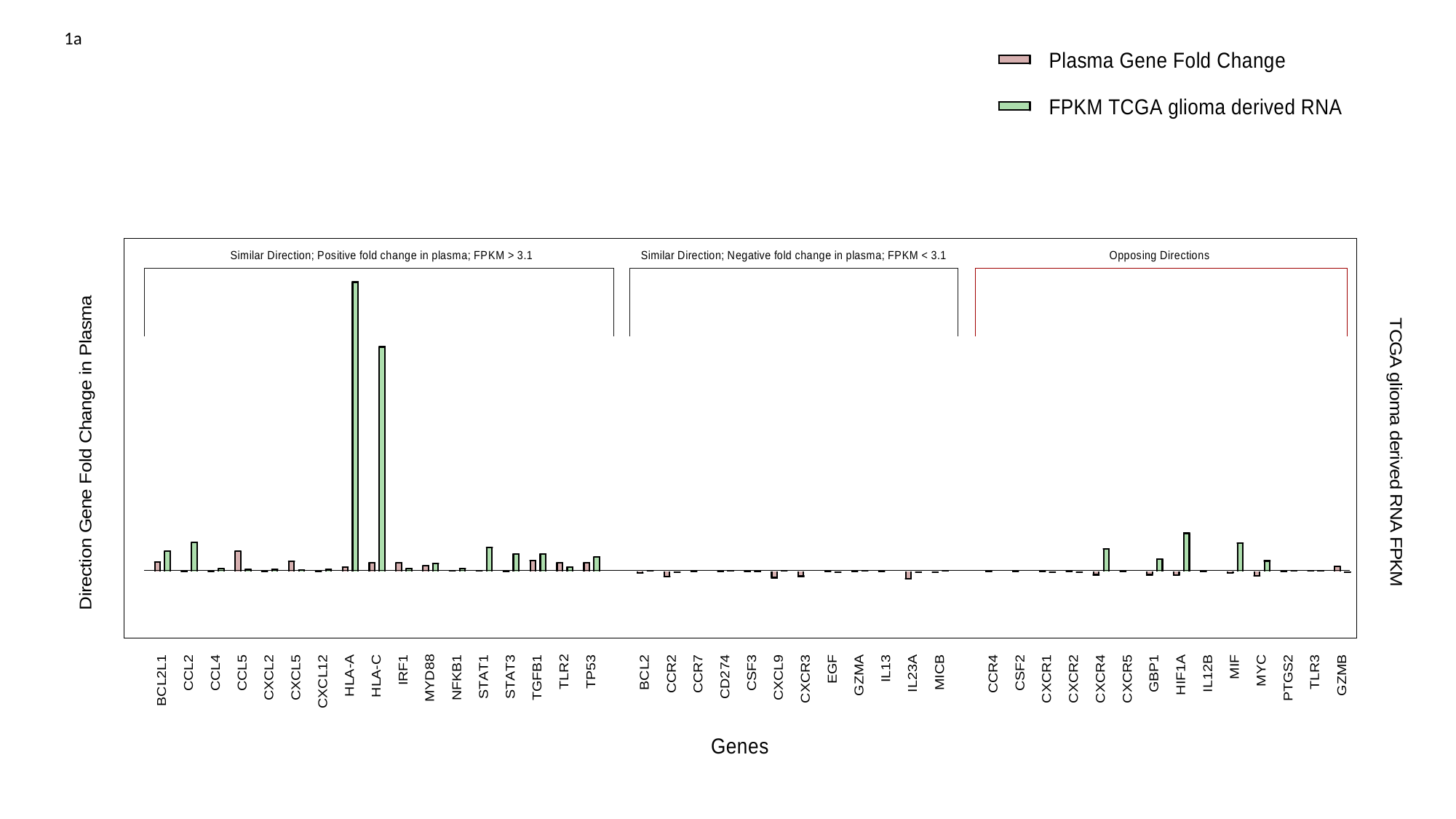

1a

Supplement: Supplementary file 1 — Supplementary figure 1a-b. (a) Data plot demonstrating the direction of fold change of amplifiable ccfmRNA transcripts in plasma samples and the mean FPKM of TCGA glioma derived RNA samples (FPKM values less than the median < 3.1 were considered to be low, and FPKM values greater than the median > 3.1 were considered to be high). (b) Data plot demonstrating no correlation between the magnitude of fold change of all amplifiable cell free messenger RNA transcripts identified in the plasma samples of glioma patients and the mean FPKM of TCGA glioma derived RNA samples (Spearman r=0.2514, n=43, p=0.104). (PPTX 49 KB) [file 701_2021_5014_MOESM1_ESM.pptx]

## Slide 1
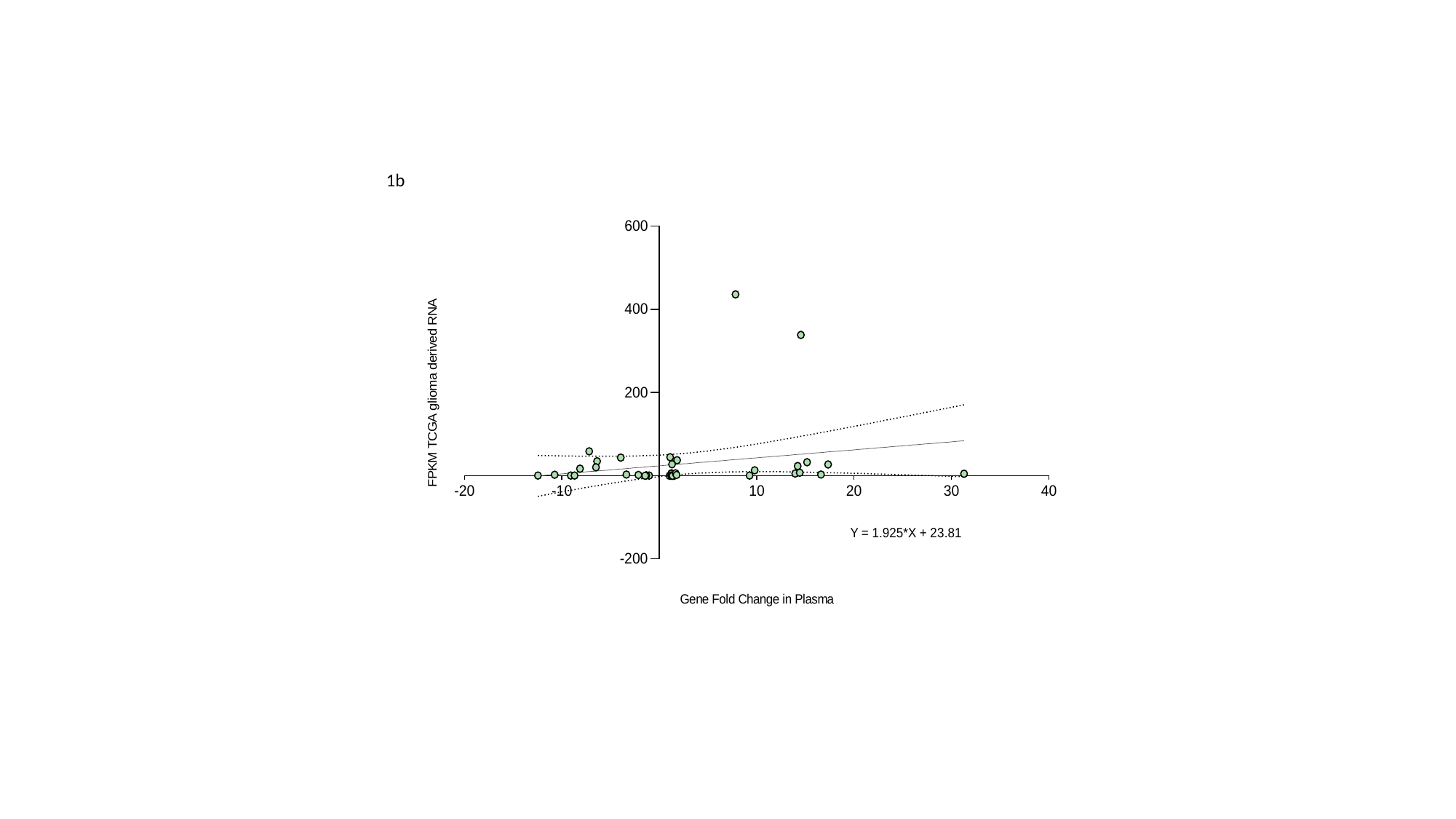

1b

Supplement: Supplementary file 2 — (PPTX 54 KB) [file 701_2021_5014_MOESM2_ESM.pptx]

## Slide 1
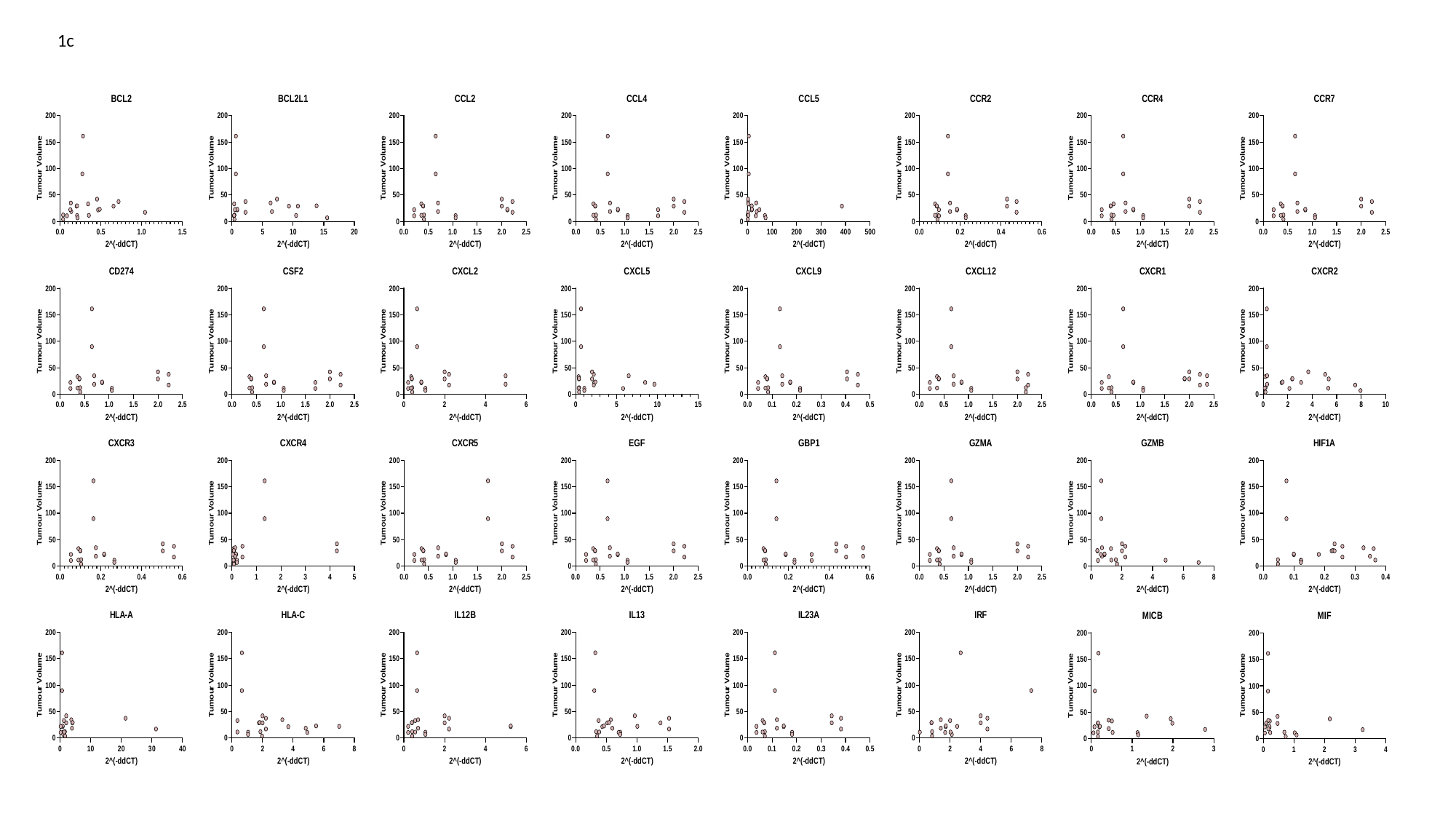

1c

Supplement: Supplementary file 3 — Supplementary figure 1c-d. Data plot showing no correlation between glioma radiographic tumour burden and the magnitude of fold change of amplifiable cell free messenger RNA transcripts identified in the plasma samples of glioma patients. (PPTX 197 KB) [file 701_2021_5014_MOESM3_ESM.pptx]

## Slide 1
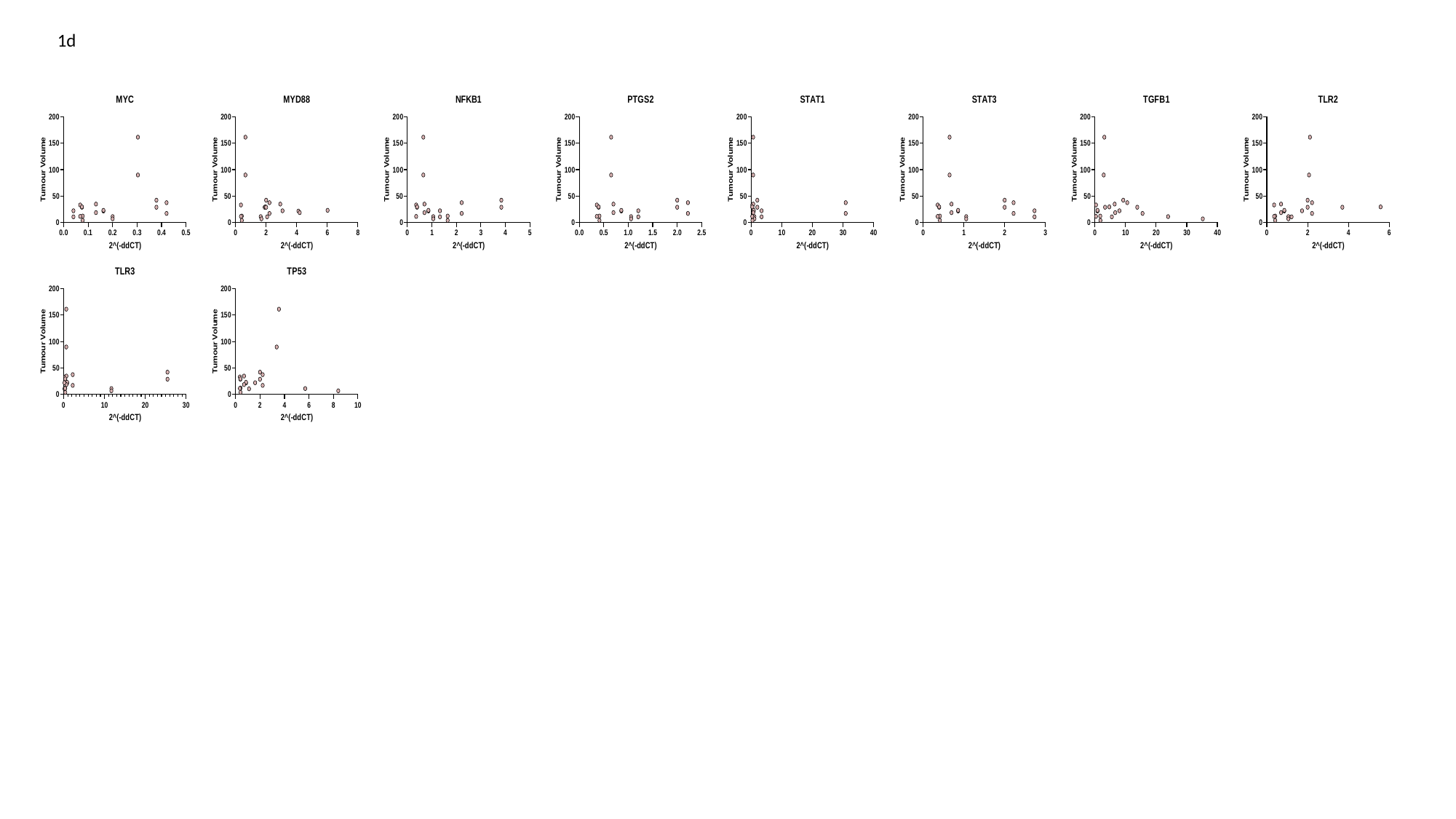

1d

Supplement: Supplementary file 4 — (PPTX 82 KB) [file 701_2021_5014_MOESM4_ESM.pptx]
